# Challenges of Using High-Dose Fractionation Radiotherapy in Combination Therapy

**DOI:** 10.3389/fonc.2016.00165

**Published:** 2016-06-30

**Authors:** Ying-Chieh Yang, Chi-Shiun Chiang

**Affiliations:** ^1^Biomedical Engineering and Environmental Sciences, National Tsing Hua University, Hsinchu City, Taiwan; ^2^Radiation Oncology, National Taiwan University Hospital Hsin-Chu Branch, Hsinchu City, Taiwan

**Keywords:** stereotactic ablative body radiation therapy, high-dose fractionation radiotherapy, chemotherapy, target therapy, immunotherapy

## Abstract

Radiotherapy is crucial and substantially contributes to multimodal cancer treatment. The combination of conventional fractionation radiotherapy (CFRT) and systemic therapy has been established as the standard treatment for many cancer types. With advances in linear accelerators and image-guided techniques, high-dose fractionation radiotherapy (HFRT) is increasingly introduced in cancer centers. Clinicians are currently integrating HFRT into multimodality treatment. The shift from CFRT to HFRT reveals different effects on the tumor microenvironment and responses, particularly the immune response. Furthermore, the combination of HFRT and drugs yields different results in different types of tumors or using different treatment schemes. We have reviewed clinical trials and preclinical evidence on the combination of HFRT with drugs, such as chemotherapy, targeted therapy, and immune therapy. Notably, HFRT apparently enhances tumor cell killing and antigen presentation, thus providing opportunities and challenges in treating cancer.

## Introduction

With advances in modern image-guided techniques and the availability of high-end linear accelerators and particle therapy, radiation oncologists can administer considerably high radiation doses per fraction to tumors, with rapid dose fall-off from the target (namely the tumor) and acceptable normal tissue toxicity. High-dose fractionation radiotherapy (HFRT) for intracranial tumors is called stereotactic radiosurgery (SRS), and that for extracranial tumors is called stereotactic ablative body radiotherapy (SABR) or stereotactic body radiotherapy (SBRT).

Some clinical conditions, such as early-stage non-small cell lung cancer (NSCLC), prostate and pancreatic cancer, and oligometastases, are potential targets for HFRT. The biological effective dose (BED) (1, 2) can be calculated using the conventional linear-quadratic (LQ) model, which fits well in conventional fractionation radiotherapy (CFRT) (fraction size, approximately 2 Gy). The hot spots within the irradiated field become hotter while converting the physical radiation dose to BED, called the “double trouble” effect. In brief, it introduces errors in physical dose measurement because of inhomogenous tissue composition, and the error is amplified with differences in doses per fraction during CFRT. Furthermore, the BED differs for different α/β ratios because of tissue heterogeneity. A higher fractionation dose of radiotherapy (RT) (typically, >8 Gy per fraction) results in an unequally distribution of BED, known as the “triple trouble” (3). Factors, such as reoxygenation, vascular endothelial cell death, and antitumor immunity, in HFRT further complicate the situation (4). Therefore, dose calculation and estimation constitute the initial challenges, in HFRT.

Clinically, cytotoxic drugs, targeted agents, or immune modulators have been combined with CFRT to improve local control and survival. This strategy can be reasonably used with HFRT in clinical trials; however, different considerations may be necessary. Herein, we review the evidence on preclinical and clinical trials that have combined HFRT with drugs.

## Chemotherapy and High-Dose Fractionation Radiotherapy

Previous studies (5–10) have reported that using concurrent chemotherapy as a radiosensitizer improves local control and prolongs the overall survival of patients with head and neck squamous cell carcinoma, uterine cervical cancer, malignant glioma, locally advanced NSCLC gastric cancer, and locally advanced rectal cancer in CFRT. However, the outcomes of administering radiosensitizing chemotherapy with HFRT remain controversial. Ohri et al. (11) analyzed clonogenic survival data from 26 studies to estimate the biologically equivalent doses in 2-Gy fractions (EQD2) (12) for HFRT with or without radiosensitizing chemotherapy in glioma, head and neck cancer, pancreatic cancer, and NSCLC cell lines using a generalized LQ model. The EQD2 is the dose equivalent to the radiation dose given in 2-Gy fractions. They concluded that combined with HFRT, radiosensitizing chemotherapy increased the EQD2 by 28–82%, depending on the disease site, and that combined with CFRT, it increased the EQD2 by 34–169%. No significant differences existed between the HFRT and CFRT groups (*p* = 0.3). However, for some diseases, such as pancreatic cancer, the EQD2 increased by 82 and 34% for HFRT and CFRT, respectively (*p* < 0.001). Thus, HFRT is apparently more effective than CFRT in chemoradiotherapy for pancreatic cancers. By contrast, head and neck cancer is frequently treated using fractionation therapy, and the 50% EQD2 increase obtained using CFRT is preferable to the 28% increase obtained using HFRT. This observation supports the position of chemoradiotherapy using CFRT as the standard treatment for locally advanced head and neck cancer. However, in a hyperfractionated and accelerated head and neck cancer clinical trial not involving HFRT (fraction dose <1.8), chemotherapy increased the BED by only approximately 10 Gy_10_, which is equivalent to the addition of 12 Gy in 2 Gy daily or 1.2 Gy twice daily (13). According to the calculation by Kasibhatla et al. (13) and correction by Fowler (14), the chemotherapeutic effect was 3.6 fractions of 2-Gy added to CFRT. This again highlights the importance of the dose per fraction and cautions the extension from the experience of combining chemotherapy with CFRT to HFRT.

Some clinical trials examining the potential of chemotherapy combined with HFRT have recently concluded or are still under way. For example, the first-ever SABR radiochemotherapy phase I trial was recently completed and identified a safe dose of carboplatin–gemcitabine chemotherapy 1 day preceding SABR for both local and regional or distant gynecologic cancer, resulting in a 79% partial response and 21% disease stability (15). Additional well-designed translational clinical trials evaluating the optimal timing and sequence are warranted; however, this trial provided substantial data on treating women with recurrent or persistent gynecologic cancer by using chemotherapy combined with HFRT. Moreover, the trial yielded encouraging results that can serve as a basis for future trials on concurrent chemotherapy and HFRT for other disease sites. Several trials have treated locally advanced pancreatic cancer with chemotherapy and HFRT and have reported it to be tolerable and promising. Three clinical trials have used 25 Gy in five fractions with neoadjuvant or adjuvant chemotherapy containing gemcitabine and reported grade 3 toxicity of <5.3% and median survival of 12.2–18.8 months (16, 17).

## Epidermal Growth Factor Receptor-Targeted Therapy and High-Dose Fractionation Radiotherapy

The advent of mechanism-based therapy promoted the development of targeted therapy for cancer treatment. Many molecular-targeted drugs have been developed to treat hematological and solid tumors that have specific driver molecular aberrations. Some clinical trials have reported clinical results superior to traditional cytotoxic chemotherapy results. For example, ZD1839 (gefitinib) as an epidermal growth factor receptor (EGFR) tyrosine kinase inhibitor was superior to the first-line carboplatin–paclitaxel regimen in East Asian patients with lung adenocarcinoma who were non-smokers or former light smokers (hazard ratio for progression or death, 0.74) and more favorable for patients with EGFR gene mutation subgroup (hazard ratio for progression or death, 0.48) (18). The objective response rate was 71.2 and 1.1% for patients with and without EGFR mutation, respectively. Erlotinib alone was also superior to the standard chemotherapy in patients with specific mutations (19).

Some molecular pathways targeted by molecular targeting drugs can compromise the pathway that leads to radioresistance. For example, EGFR inhibitors, such as gefitinib, erlotinib, and cetuximab, can prevent the radiation-induced autophosphorylation of EGFR proteins and downstream substrates, such as the DNA-PK enzyme involved in DNA damage repair. Therefore, combined modality treatment, such as chemoradiotherapy, is a rational means of improving local control and survival. HFRT combined with the anti-EGFR blocking antibody, C225, has been shown to have synergistic or additive effects *in vitro* by inhibiting the antiapoptotic proteins Bcl-xl and Bacl-2, as well as the phosphorylated form of Akt protein, transforming growth factor α, vascular endothelial growth factor (VEGF), and basic fibroblast growth factor (20–22). The *in vivo* efficacy of C225 was also illustrated in a preclinical animal model, proving the potential of EGFR inhibitor combined with HFRT. For example, the combination of gefitinib and HFRT (10 Gy × 4 fractions) resulted in long-term survival of 10% of tumor-bearing mice (21). Notably, the synergistic effect depends on driver mutation, which in this case is EGFR mutation (22). Furthermore, several clinical trials have suggested that EGFR inhibitor combined with CFRT is well tolerated and effective in several solid tumors, such as those of head and neck cancer (23, 24), NSCLC (25), rectal cancer (26), and esophageal squamous cell carcinoma (27). These are positive drivers for trials to determine the efficacy of targeted therapy combined with HFRT.

Phase II clinical trials have reported the safety and efficacy of concurrent cetuximab and HFRT for locoregional pre-irradiated or head and neck cancers in elderly patients (28, 29). However, additional randomized studies on targeted molecule therapy combined with HFRT are warranted to further confirm the benefits of this regimen at different disease sites or with different combination sequences.

## VEGF-Targeted Therapy and High-Dose Fractionation Radiotherapy

Vascular endothelial growth factor is a proangiogenic agent that directly stimulates the vascular endothelial cells and initiates neovasculature (30). It is also an immune factor that could impair the function and maturation of dendritic cells, which could be reversed by a VEGF blockade (31, 32). In contrast to other targeted therapies, anti-VEGF therapy does not target an oncogene-driven mutation. Bevacizumab prolongs median survival by 4.7 months for metastatic colorectal cancer (33). Two phase III studies (AVAglio and RTOG 0825) have administered bevacizumab combined with a standard treatment (surgery followed by CFRT and oral temozolomide) for newly diagnosed glioblastoma (GBM) and reported progression-free survival; however, overall survival was similar between treatment and placebo arms (34, 35). Furthermore, in a phase II GLARIUS trial of patients with newly diagnosed *O*^6^-methylguanine-DNA methyltransferase non-methylated GBM on whom temozolomide had limited efficacy, CFRT with concurrent and adjuvant bevacizumab plus irinotecan instead of temozolomide increased 6-month progression-free survival by 36.7% (79.3–42.6%); similar to the aforementioned trials, overall survival was similar (36).

High-dose fractionation radiotherapy combined with anti-VEGF therapy demonstrates a synergistic effect, which is related to many possible mechanisms. First, anti-VEGF therapy increases the pO_2_ level to compensate for the radioresistance of hypoxic tumors (37). Although RT frequently reduces tumor vessel density and tumor blood flow, the oxygen concentration can be increased by reducing interstitial fluid pressure and killing oxygenated cells (38). Second, anti-VEGF agents could prevent VEGF-induced angiogenesis after HFRT (39). Third, the VEGF also protects the endothelial cells from radiation; therefore, HFRT (8 × 3 Gy) combined with anti-VEGF therapy exerts a synergistic effect on endothelial cell killing (40). Fourth, the optimal-dose of an anti-VEGF agent not only increases immune cell (DC and CD8^+^ cells) infiltration and anticancer immune response (41) but also normalizes the vascular network to enhance the efficacy of HFRT. A vascular “normalization window” has been reported to appear approximately 2–5 days after the administration of an anti-VEGF agent, depending on the tumor type and disease site. This “window” is associated with the increase of pericyte coverage and angiopoietin-1 (Ang1) expression and thinning of basement membranes, resulting in enhanced tumor oxygenation. This can also be the window for administering HFRT or cytotoxic agents (42, 43). However, the side effects of this approach have not been comprehensively evaluated. Bevacizumab combined with chemotherapy for advanced NSCLC increases side effects, including lethal pulmonary hemorrhage (44). The incidence of severe bleeding events was 3% in a phase IV trial (45). A phase I trial of concurrent CFRT and bevacizumab for stage III NSCLC was terminated because of additional radiation pneumonitis (46). When CFRT, capecitabine, and bevacizumab are combined for inoperable pancreatic adenocarcinoma, tumor involvement of duodenal mucosa causes ulceration and bleeding (47). Two clinical phase II trials of chemoradiotherapy plus bevacizumab were terminated early because of severe toxicities of the tracheoesophageal fistulae (48). This emphasizes the importance of carefully designing clinical trials for combining HFRT with anti-VEGF therapy.

## Phosphatidylserine Targeting Therapy and High-Dose Fractionation Radiotherapy

Microvascular endothelial apoptosis is pivotal in controlling a tumor with a fractionation size larger than 10 Gy (49). Studies have reported that the acid sphingomyelinase (ASMase) can translocate to cell membranes and convert sphingomyelin into ceramide. Ceramide activates apoptotic protein BAX to release the mitochondrial cytochrome *c*, which triggers the intrinsic apoptotic pathway to mediate the cytotoxic effect of high-dose radiation on endothelial cells (50, 51).

The vascular endothelium of GBM and many solid tumors, but not of normal tissues, expresses phosphatidylserine (PS). RT could further increase the exposure of PS in the tumor vessel endothelium. HFRT plus anti-PS antibodies (bavituximab) further damaged the tumor vasculature in a murine glioma model, resulting in increased tumor control (52). HFRT induces the binding of an anti-PS antibody to PS on the cell surface and, subsequently, leads to antibody-dependent killing of endothelial cells. The long-term survival for tumor-bearing mice after combination therapy was resistant to the rechallenge of F98 glioma cells. This suggests the potential for clinical trials of bavituximab combined with HFRT for patients with GBM (52). In addition, radiation-enhanced PS exposure further enhances the efficacy of glioma therapy by activating a soluble tissue factor to trigger the extrinsic coagulation cascade, thus causing selective thrombosis of GBM vasculature (53). These findings indicate that HFRT has a synergistic potential when combined with vascular targeting therapy.

A phase I clinical trial of bavituximab plus paclitaxel for metastatic breast cancer was well tolerated and yielded promising results (54). Pandya et al. (55) reported trials conducted using gemcitabine with and without bavituximab for metastatic pancreatic adenocarcinoma. Gemcitabine combined with bavituximab did not improve the survival or overall response rate (56). Additional trials on bavituximab plus CFRT are underway.

## Hyperbaric Oxygenation Therapy and High-Dose Fractionation Radiotherapy

Most solid tumors have tortuous and dilated microvessels with loose pericyte coverage and increased interstitial fluid pressure, resulting in heterogeneous hypoxic areas within the tumor. Tumor hypoxia is related to radiation resistance and poor prognosis (57). The hypofractionation associated with HFRT can exert more therapeutic effects by decreasing the possibility of tumor repopulation, but could also be compromised by the increase of tumor hypoxia (58). Toma-Dasu et al. used *in silico* tumor models with heterogeneous oxygenation and reported that hypoxia reduces tumor control probability after single-fraction RT, particularly in larger tumors. Local reoxygenation by four or five fractionations could partially reverse the effect of hypoxia (59), supporting the clinical trials of HFRT that favored single-fraction RT.

Hyperbaric oxygenation (HBO) directly relieves the tumor hypoxia in patients with head and neck cancer and GBM (60, 61). Overgaard systemically reviewed 32 randomized clinical trials on hypoxic modifiers, such as normobaric oxygen or carbogen breathing, HBO, and hypoxic radiosensitizers, and observed that these modifiers were all effective in locoregional control of head and neck squamous cell carcinoma. These hypoxic modifiers benefit not only CFRT but also high-dose HFRT considering locoregional control and disease-free survival (62). Despite the practical difficulty and inconvenience of concurrent combination of HBO with HFRT, several trials have reported positive results of administering CFRT immediately after HBO (63). For example, HBO combined with CFRT for patients with malignant glioma yielded a higher response rate and improved the median survival from 12 to 24 months. All the patients in the HBO group received irradiation within 15 min following HBO (64). HBO combined with CFRT for uterine cervical cancer also improved local control and survival (65).

In addition, HBO therapy is safe and effective against radiation-related tissue damage or necrosis such as mandibular osteoradionecrosis as well as radiation proctitis and cystitis (66). The prophylactic use of HBO within 1 week following single-fraction RT for brain metastases reduced the incidence of radiation necrosis or white matter injury from 20 to 10% (67). Kohshi et al. reported that the administration of gamma HFRT immediately following HBO therapy has survival benefits for patients with recurrent glioma (64). The dual benefits of HFRT combined with HBO therapy provide a promising direction for further investigation.

## Immune Therapy and High-Dose Fractionation Radiotherapy

The development of cancer immunity is a cycle with stepwise events that require (1) releasing tumor-associated specific antigens, (2) presenting cancer antigens, (3) priming and activating antigen-presenting cells (APCs) and T cells, (4) recruiting cytotoxic T cells to tumors, (5) infiltrating T cells into tumors, (6) recognizing cancer cells, and (7) killing cancer cells, thus releasing tumor antigens that feed back to the first step of this cycle (68). A vaccine for cancer has been anticipated to have effects similar to those against infectious diseases (e.g., bacterial or virus infection). However, it alone involves only the first three steps of the aforementioned cycle and yields limited clinical results because it is difficult to generate potent cytotoxic T cell responses against cancer cells, to correct the immunosuppressive microenvironment, and to prevent the immunoediting effect of cancer cells.

The efficacy of RT was found to depend not only on radiobiological factors but also on the immunological competence of the host (69); therefore, RT was immediately recognized as a potential immune boosting agent for developing anticancer immunity. Milas et al. (70) reported that local irradiation enhanced the efficacy of the antitumor immune response of *Corynebacterium granulosum* and *Cryptosporidium parvum* bacteria in a murine fibrosarcoma tumor model. Many preclinical and clinical trials have examined the potential of RT combined with immunotherapy in various cancer models. RT, particularly HFRT, plays several roles in tumor immunity. Radiation not only kills tumor cells to release antigens and induces stromal cells and vascular endothelial cells to produce immune-associated factors but also eliminates APCs and T cells (71). However, in contrast to the systemic effects of cytotoxic chemotherapy, radiation-induced killing of immune cells is localized to the tumor region (71). Radiation can also promote protein degradation and increase the cell surface expression of major histocompatibility complex-I with a dose-dependent presentation of endogenous peptides (72). A study showed that the activity of transporter-associated antigen presentation lasted longer with 25 Gy than with lower doses (73). Compared with five fractions (5 × 3 Gy), HFRT (in this case, 1 × 15 Gy) further increased the APCs carrying tumor antigens in tumor-draining lymph nodes where the tumor antigen-reactive and TNF-γ-secreting T cells were also increased. Those antitumor T cells had an increased ability to migrate to and infiltrate the tumors on day 14 (74). These promising preclinical studies have prompted trials on combining immunotherapy with RT; however, the results of such clinical trials have not been as promising as preclinical studies despite tolerance and safety being acceptable. This is mainly because radiation, in addition to being a promising immunological adjuvant, is a complex modifier of the tumor microenvironment. Irradiation not only induces prevailing antitumor immunity but also activates immuneosuppressive pathways (75). The balance shift between radiation-induced immune activation and suppression depends not only on the disease sites but also on the dose per fraction applied and the total dose (76).

## Immune Checkpoint Blockade and High-Dose Fractionation Radiotherapy

Cancer immunotherapy was considered a revolution for patients with cancer. Research focused on developing therapeutic vaccines using T cells in various approaches, including those involving whole tumor cells expressing cytokines and DNA vaccines, or antigen-pulsed dendritic cell therapy. Despite researchers expressing enthusiasm in the 1990s, most of them failed to show objective clinical responses, and the enthusiasm waned by the late 1990s (77). One major reason for the failure of earlier cancer immunotherapy trials was possibly unawareness about the other side of T cell activation, the inhibitory program mediated by immune checkpoints, such as cytotoxic T lymphocytes-associated protein 4 (CTLA-4) or programed death-1 (PD-1) protein. This shifted the strategies for cancer immunotherapy from activating T cells to unleashing them (78). By targeting the inhibitory pathways in T cells to reverse the suppressed antitumor T cell response, the immune checkpoint blockade therapy approach yields many favorable clinical results, recently (78). Anti-CTLA-4 antibodies (ipilimumab or tremelimumab) and anti-PD-1 antibodies (Nivolumab or Pembrolizumab) are currently approved by the U.S. Food and Drug Administration (FDA) for immune checkpoint blockade therapy.

Clinical trials have proposed the clinical use of immune checkpoint inhibitors, and favorable responses by using immunotherapy have been elicited in metastatic melanoma (79), NSCLC (80, 81), renal cell carcinoma (80), bladder cancer (82), and head and neck cancer (83); however, responses are limited to patients with relatively immunogenic tumors or some degree of preexisting tumor-infiltrating T cells. Combination treatment is considered more appropriate for most patients with cancer. Combining immune checkpoint inhibitors with various modalities, such as surgery, chemotherapy, targeted therapy, vaccine or immune therapy, or RT, was initiated in many preclinical models and clinical trials (78). The main rationale for this strategy is likely that one component of combination therapy can reduce the tumor burden or tumor-associated immunosuppression and subsequently enhance the induction of tumor immunity and the development of long-term immune memory. Among these combination strategies, RT has been long considered as the ideal partner for immune checkpoint inhibitors because radiation has several pro-immunogenic effects (76), such as the release of tumor antigens, activation of canonical immune pathways, and generation of immune active tumor microenvironments, which improve the response to immune checkpoint inhibitors (84). Notably, local tumor irradiation may produce tumor regression at a distant site, referred to as the abscopal effect. However, such observations are rare (85). With conventional fractionation, only 10 patients exhibiting the abscopal effect have been reported, since 1973 (85); their primary sites have included melanoma, renal cell carcinoma, and hepatocellular carcinoma. With HFRT (8–26 Gy per fraction), the abscopal effect has been reported in five cases of renal cell carcinoma, one case of NSCLC, and two cases of melanoma, since 2006 (86–90). These occasional radiation-induced abscopal responses indicate that local tumor irradiation may function as an *in situ* tumor vaccine.

Demaria et al. (91) were the first to reveal that local irradiation at a tumor resistant to the CTLA-4 blockade therapy could render it sensitive to anti-CTLA-4 antibody therapy and inhibit metastasis in a murine breast tumor model. They further demonstrated that CTLA-4 blockade combined with fractionated (3 × 8 Gy), but not single-dose (1 × 20 Gy), RT develops abscopal responses (92). These responses of ipilimumab combined with RT have since been confirmed in several clinical reports in patients with melanoma unresponsive to the CTLA-4 inhibitor as single modality (88, 89, 93). These preclinical and clinical results not only demonstrate that RT potentially can function as an *in situ* tumor vaccine but also indicate the crucial role of RT treatment protocols. Several ongoing clinical trials are determining the benefits of RT combined with anti-CTLA-4 therapy (94).

The inhibition of the PD-1/PD-L1 pathway is the second blockade strategy proved by the FDA for immune checkpoint blockade therapy. Many preclinical models have shown that this approach promotes host CTL expansion and results in tumor regression (95–98). These preclinical results have also been successfully illustrated in several clinical reports (80, 99); however, similar to the CTLA blockade, the positive response is still limited to certain patients. A combination therapy has been considered for expanding the responder proportion of PD-1 blockade therapy, and the combination with RT is being investigated in several laboratories (94). Zeng et al. (100) reported that anti-PD-1 therapy combined with stereotactic RT (1 × 10 Gy) significantly prolongs the survival of glioma tumor-bearing mice and generates long-term antitumor memory. In another report, Dovedi et al. (101) demonstrated that low doses of CFRT (5 × 2 Gy) increased PD-1 expression in tumors for 7 days immediately after the RT exposure. Separately combining the αPD-L1 monoclonal antibody (mAb) and αPD-1 mAb with RT cured 66 and 80% of mice, respectively, whereas immune therapy alone did not improve survival (101). This curative effect was associated with the development of CD8^+^ T cell responses, which protects surviving mice against tumor rechallenge. Moreover, Deng et al. (75) demonstrated that HFRT locally controls tumors through direct cell killing and boosts tumor-specific immunity to suppress the growth of both local and distant tumors. However, this radiation-induced antitumor immunity is insufficient for destroying tumors, and the tumor frequently regrew because of PD-L1 upregulation by tumor and myeloid cells and PD-1 downregulation by CD8^+^ T cells. This study indicates that irradiation not only generates an *in situ* antitumor vaccine but also inhibits the T cell function as an effect on the alteration of the PD-L1/PD-1 axis in the tumor microenvironment. This study provides a rational design for increasing the antitumor immunity of HFRT through PD-L1 blockade or regulator T cell (Treg) depletion (102).

## Conclusion

In the past 20 years, technological advances have considerably changed the delivery of radiation and enabled radiation oncologists to safely deliver higher radiation doses to tumors. This change not only increases the cytotoxic power of radiation but also provides new avenues for improving cancer therapy. This new opportunity is largely attributed to the change related to the tumor microenvironment following HFRT. Compared with CFRT, tumor microenvironmental factors, such as tumor hypoxia, T-cell immune response, tumor vasculature, and cytokines, are all different following HFRT. The situation is more complex when HFRT is combined with chemotherapy, targeted therapy, or an immune checkpoint blockade. It is promising that HFRT as neoadjuvant therapy can initially kill tumor cells, enhance antigen presentation, and promote T-cell immune response, thereby optimizing the immune checkpoint blockade treatment. The new option for HFRT provides an opportunity for and a challenge in treating cancers (76).

## Author Contributions

Y-CY wrote the manuscript. C-SC advised and refined this manuscript.

## Conflict of Interest Statement

The authors declare that the research was conducted in the absence of any commercial or financial relationships that could be construed as a potential conflict of interest.
